# Ten years of treatment with ruxolitinib for myelofibrosis: a review of safety

**DOI:** 10.1186/s13045-023-01471-z

**Published:** 2023-07-27

**Authors:** Srdan Verstovsek, Ruben A. Mesa, Robert A. Livingston, Wilson Hu, John Mascarenhas

**Affiliations:** 1 Leukemia Department, The University of Texas MD Anderson Cancer Center, 1515 Holcombe Blvd, Houston, TX 77030 USA; 2 Atrium Health Wake Forest Baptist Comprehensive Cancer Center, Wake Forest University School of Medicine, Medical Center Blvd, 11th Floor, Winston-Salem, NC 27157 USA; 3 Incyte Corporation, 1801 Augustine Cut-Off, Wilmington, DE 19803 USA; 4 Icahn School of Medicine at Mount Sinai, 1470 Madison Avenue, New York, NY 10029 USA

**Keywords:** Janus kinase, Myelofibrosis, Myeloproliferative neoplasm, Ruxolitinib, Safety

## Abstract

Myelofibrosis (MF) is a chronic myeloproliferative neoplasm characterized by bone marrow fibrosis, anemia, extramedullary hematopoiesis, and splenomegaly. Patients with MF are at risk for reduced survival versus the general population and often experience burdensome signs and symptoms that reduce quality of life. The oral Janus kinase (JAK) 1/JAK2 inhibitor ruxolitinib was initially approved by the US Food and Drug Administration in 2011 for the treatment of patients with intermediate or high-risk MF, including primary MF, post-polycythemia vera MF, and post-essential thrombocythemia MF, based on efficacy and safety findings from the randomized, controlled, phase 3 COMFORT trials. Over a decade later, ruxolitinib continues to be the standard of care in higher-risk MF, and dose optimization and management remain crucial for safely maximizing clinical benefits of ruxolitinib. This review summarizes the safety profile of ruxolitinib in patients with MF in the COMFORT trials leading up to approval and in the subsequent JUMP, ROBUST, EXPAND, and REALISE trials; in pooled analyses; and in postmarketing analyses in the 10 years following approval. There is a focus on the occurrence of common hematologic and nonhematologic adverse events, with guidance provided on the management of patients with anemia or thrombocytopenia, including dosing strategies based on findings from the REALISE and EXPAND trials. Finally, to ensure a greater understanding of the safety profile of ruxolitinib, practical considerations are discussed.

**Supplementary Information:**

The online version contains supplementary material available at 10.1186/s13045-023-01471-z.

## Introduction

**Fig. 1:**
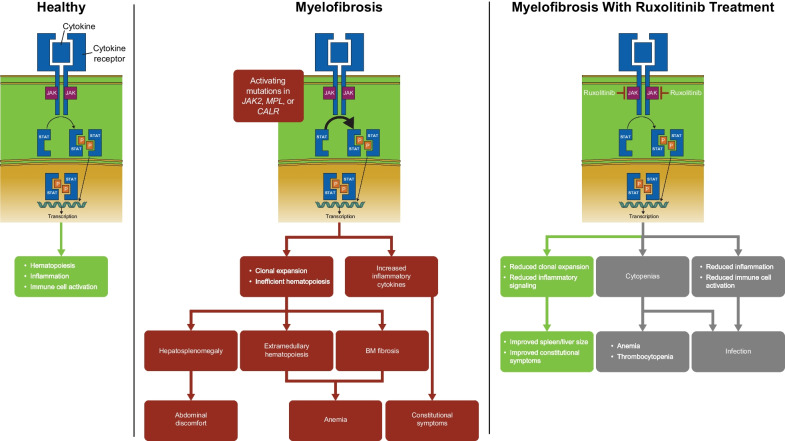
JAK signaling in MF and ruxolitinib mechanism of action. *BM* bone marrow, *JAK* Janus kinase, *MF* myelofibrosis, *P* phosphorylation, *STAT* signal transducer and activator of transcription

Ruxolitinib is an oral Janus kinase (JAK) 1/JAK2 inhibitor first approved by the US Food and Drug Administration (FDA) in 2011 for the treatment of adults with intermediate or high-risk myelofibrosis (MF), including primary MF, post-polycythemia vera (PV) MF, and post-essential thrombocythemia MF (Fig. 1) [1]. The approval of ruxolitinib was based on efficacy and safety demonstrated in the randomized, placebo-controlled phase 3 COMFORT trials [2, 3]. Subsequently, ruxolitinib was approved for PV in patients with inadequate response to or intolerance of hydroxyurea (HU), steroid-refractory acute graft-versus-host disease (GVHD), and chronic GVHD after failure of systemic therapy [1]. Ruxolitinib has been shown to not only improve splenomegaly and the burdensome symptoms associated with MF but also to improve overall survival (OS) [2–4]. Consequently, more than a decade since its initial approval, ruxolitinib continues to be the standard of care in patients with higher-risk MF [5]. During this time, much has been learned about the tolerability of ruxolitinib, as well as dosing and management strategies to safely maximize clinical benefit.

Here we review the safety profile of ruxolitinib, taking into account patients with MF who participated in clinical trials (including the pivotal COMFORT [2, 3], phase 2 UK-based ROBUST [6], and expanded-access phase 3b JUMP [7] trials; the dose-finding phase 1b EXPAND [8] trial in patients with thrombocytopenia; and the phase 2 REALISE [9] trial in patients with anemia) as well as those evaluated in postmarketing studies. We also review previously unpublished postmarketing safety surveillance data collected during the 10 years following initial FDA approval.

## Overview of commonly reported adverse events

### Hematologic adverse events: clinical trial experience

Cytopenias, in particular anemia and thrombocytopenia, are the most frequent adverse events (AEs) with ruxolitinib in patients with MF (Table 1) [2, 3]. This outcome is unsurprising based on inhibition of JAK2, which regulates thrombopoietin and erythropoietin signaling [10, 11]. In the pivotal phase 3 COMFORT-I study (ClinicalTrials.gov number, NCT00952289), about half of the grade 3 or 4 anemia and thrombocytopenia AEs occurred during the first 8 weeks of treatment [3]. These AEs were generally manageable with dose modifications, transfusions, or both, with only two patients randomized to ruxolitinib and two randomized to placebo discontinuing treatment due to anemia or thrombocytopenia (n = 1 each for each study treatment group). Importantly, although mean hemoglobin and platelet levels decreased during the first 8–12 weeks of treatment, both stabilized thereafter, with hemoglobin levels increasing toward baseline before stabilizing [4]. Similar findings were reported in COMFORT-II (NCT00934544) [2]. Grade ≥ 3 or serious AEs related to bleeding occurred infrequently regardless of study treatment [2, 3, 12].

**Table 1:** Key safety data from ruxolitinib MF trials

Event	COMFORT-I [15, 30]			COMFORT-II [2, 12]			JUMP [7]	ROBUST [6]	EXPAND [8]		REALISE [9]
	RUX (n = 155)	PBO (n = 154)	RUX Crossover (n = 111)	RUX (n = 146)	BAT (n = 73)	RUX Crossover (n = 45)	RUX (n = 2233)	RUX (n = 48)	PLT 75–99 × 10^9^/L (n = 20)	PLT 50–74 × 10^9^/L (n = 18)	RUX (n = 51)
Main trial results, * %											
Anemia											
All grade	96	87	—	97^†^	90^†^	—	59	46	45	44	35
Grade 3/4	45	19	—	42^†^	30^†^	—	35	21	20	17	31
Thrombocytopenia											
All grade	70	31	—	68^‡^	27^‡^	—	53	38	40	78	29
Grade 3/4	13	1.3	—	8.2^‡^	6.8^‡^	—	19	13	35	78	20
Herpes zoster											
All grade	NR	NR	—	NR	NR	—	5.2	0	NR	NR	NR
Grade 3/4	NR	NR	—	NR	NR	—	0.5	0	NR	NR	NR
Sepsis											
All grade	NR	NR	—	NR	NR	—	NR	4.2^‖^	NR	NR	NR
Grade 3/4	NR	NR	—	NR	NR	—	1.5	4.2^‖^	NR	NR	NR^#^
TB											
All grade	NR	NR	—	NR	NR	—	0.2	0	NR	NR	NR
Grade 3/4	NR	NR	—	NR	NR	—	0.04	0	NR	NR	NR
Grade 3/4 PML	NR	NR	—	NR	NR	—	0	2.1	NR	NR	NR
Grade 5 MACE										—	
Cardiac arrest	NR	NR	NR	NR	NR	NR	0.6	0	5.0	0	0
MI	NR	NR	NR	NR	NR	NR	0.2	0	0	0	0
Cardiac failure	NR	NR	NR	NR	NR	NR	0.5	0	0	0	2.0
Cardiopulmonary failure	NR	NR	NR	NR	NR	NR	0.3	0	0	0	0
Cardiogenic shock	NR	NR	NR	NR	NR	NR	0.2	0	0	0	0
Congestive cardiac failure	NR	NR	NR	NR	NR	NR	< 0.1	0	0	0	0
Cardiac disorder	NR	NR	NR	NR	NR	NR	< 0.1	0	0	0	0
5-year data, %											
Sepsis	NR	NR	—	7.9^§^	NR	—	NR	NR	NR	NR	NR
TB	NR	NR	—	1.0^§^	NR	—	NR	NR	NR	NR	NR
Grade 3/4 MACE,											
Congestive cardiac failure											
After 48 mo of treatment	6.2	NR	NR	NR	NR	NR	NR	NR	NR	NR	NR
Before 48 mo of treatment	0–1.1	NR	NR	NR	NR	NR	NR	NR	NR	NR	NR
MI											
After 48 mo of treatment	0	NR	NR	NR	NR	NR	NR	NR	NR	NR	NR
Before 48 mo of treatment	0–2.7	NR	NR	NR	NR	NR	NR	NR	NR	NR	NR
Grade 5 MACE											
Cardiac arrest	0.6	0	0	0.7	0	0	NR	NR	NR	NR	NR
MI	0.6	0	0.9	0.7	0	0	NR	NR	NR	NR	NR
Cardiac failure	0	0	0.9	0.7	1.4	2.2	NR	NR	NR	NR	NR
Cardiopulmonary failure	0	0	0	0.7	0	0	NR	NR	NR	NR	NR
Cardiogenic shock	0	0	0	0	0	0	NR	NR	NR	NR	NR
Congestive cardiac failure	0	0	1.8	0	0	0	NR	NR	NR	NR	NR
Cardiac disorder	0	0	0	0	0	0	NR	NR	NR	NR	NR
5-year, per 100 PYE											
Infections											
Herpes zoster											
All grade	3.5	1.0	5.8	3.9^§^	0^§^	6.3^§^	NR	NR	NR	NR	NR
Grade 3/4	0	0	0.4	NR	NR	NR	NR	NR	NR	NR	NR
Sepsis											
All grade	1.7	1.9	1.5	NR	NR	NR	NR	NR	NR	NR	NR
Grade 3/4	1.7	1.0	1.5	NR	NR	NR	NR	NR	NR	NR	NR
NMSC	NR	NR	NR	6.1^§^	3.0^§^	NR	2.7^¶^				
BCC											
All grade	2.7	3.9	4.0	NR	NR	NR	NR	NR	NR	NR	NR
Grade 3/4	0.4	0	0.8	NR	NR	NR	NR	NR	NR	NR	NR
SCC											
All grade	1.9	1.0	1.1	NR	NR	NR	NR	NR	NR	NR	NR
Grade 3/4	0.6	0	0.4	NR	NR	NR	NR	NR	NR	NR	NR

*BAT* best available therapy, *BCC* basal cell carcinoma, *MACE* major adverse cardiovascular event, *MF* myelofibrosis, *MI* myocardial infarction, *NMSC* nonmelanoma skin cancer, *NR* not reported, *PBO* placebo, *PLT* platelet, *PML* progressive multifocal leukoencephalopathy, *PYE* patient-years of exposure, *RUX* ruxolitinib; SCC, squamous cell carcinoma; TB, tuberculosis

^*^ Week 24 for COMFORT-I/II, median ruxolitinib exposure of 12.4 mo for JUMP, median ruxolitinib exposure of 13.6 mo for ROBUST

^†^Reported as hemoglobin abnormality

^‡^Reported as platelet count abnormality

^§^RUX group included the randomized and extension phase, BAT group included only the randomized phase, RUX crossover group included only the extension phase

^¶^Percentage of patients; median follow-up was 13.8 mo

^‖^Staphylococcal sepsis

^#^Grade 4 sepsis led to treatment discontinuation in 1 patient. An additional 4 (7.8%) patients died from infections and infestations

The much larger global, expanded-access JUMP trial (N = 2233 patients) [7] subsequently confirmed the hematologic AE findings reported in the COMFORT trials [2, 3]. Anemia and thrombocytopenia were the most common any-grade and grade 3/4 AEs, with anemia of any grade reported in 59.5% (grade 3/4, 34.8%) and thrombocytopenia in 53.5% (grade 3/4, 19.3%) of patients. As expected, there was a higher incidence of thrombocytopenia in patients with a low baseline platelet count (< 100 × 10^9^/L; any grade, 73.2%; grade 3/4, 54.3%); in this subgroup, rates of anemia were similar to rates in the overall study population, and no grade ≥ 3 hemorrhagic events were considered related to treatment by the investigator [7]. As observed in the COMFORT trials, anemia and thrombocytopenia generally occurred within the first 12 weeks of treatment (median time to hemoglobin nadir, 8–12 weeks; median time to platelet nadir, 4 weeks). Furthermore, these events were generally manageable with dose reductions or interruptions (occurring in 67.4% and 27.2% of patients overall, respectively), with 2.0% of patients discontinuing treatment due to anemia and 3.4% due to thrombocytopenia (2.2% and 10.1%, respectively, in those with a low baseline platelet count). Even for high-risk patients, only 3.1% discontinued due to anemia and 6.2% due to thrombocytopenia, again indicating the manageable nature of these events. These results were corroborated by findings from ROBUST [6]. Likewise, EXPAND confirmed similar levels of anemia and risk of worsening thrombocytopenia in patients with low baseline platelet counts (75–99 × 10^9^/L: any grade, 40%; grade 3/4, 35.0%; 50–74 × 10^9^/L: any grade, 77.8%; grade 3/4, 77.8%), with treatment discontinuation due to thrombocytopenia reported in only one patient (5.0%) in the 75–99 × 10^9^/L group and three patients (16.7%) in the 50–74 × 10^9^/L group. Dose modifications occurred in 30.0% and 61.1% of patients, respectively, with thrombocytopenia being the most frequent AE necessitating dose adjustment [8]. Real-world evidence in smaller patient populations also supports these overall clinical findings, with anemia and thrombocytopenia typically occurring in the first 3 months of treatment, often managed via dose modifications, and seldom requiring discontinuation of ruxolitinib [13, 14].

### Nonhematologic AEs: clinical trial experience

In COMFORT-I, the overall nonhematologic AE rate was generally similar between ruxolitinib and placebo, and nonhematologic AEs were predominantly grade 1 or 2 [3]. Ecchymosis (18.7% vs 9.3%), dizziness (14.8% vs 6.6%), and headache (14.8% vs 5.3%) occurred more frequently with ruxolitinib. In COMFORT-II, few grade 3/4 nonhematologic AEs were reported in patients treated with ruxolitinib or best available therapy [2]. Overall, the incidence of new-onset nonhematologic events stabilized or decreased with longer-term ruxolitinib treatment [12, 15].

The JUMP trial subsequently confirmed that nonhematologic AEs were predominantly grade 1 or 2, with pyrexia (any grade, 16.0%; grade 3/4, 2.4%), asthenia (any grade, 15.4%; grade 3/4, 2.1%), diarrhea (any grade, 12.5%; grade 3/4, 1.1%), and fatigue (any grade, 10.0%; grade 3/4, 1.0%) the only nonhematologic AEs occurring in more than 10% of patients [7]. The results in ROBUST, REALISE, and EXPAND were consistent with JUMP, with nonhematologic AEs predominantly grade 1 or 2 [6, 8, 9]. Similar nonhematologic AEs were reported in > 10% of patients, along with abdominal pain (27% and 24%) and epistaxis (27% and 13%), which were commonly reported in ROBUST and EXPAND, respectively. Real-world experience generally aligned with these previous findings [14, 16].

Although classified as AEs, observed increases in body weight and cholesterol levels may be beneficial in this patient population, up to a point, given that cachexia and a hypercatabolic state are common in MF and are associated with poorer prognosis [17]. Mean body weight increased in the ruxolitinib arm but decreased in the placebo arm by Week 24 (3.9 vs − 1.9 kg), with body weight stabilizing by Week 36 in the ruxolitinib arm (mean increase of 5.7 kg at Week 96) in COMFORT-I [18]. Median total cholesterol levels increased to approximately 150 mg/dL by Week 4 in the ruxolitinib arm, stabilizing thereafter at 150 mg/dL and remaining below the study upper limit of 240 mg/dL for most patients. Similarly, in JUMP, weight increase was reported for 6.3% of patients (grade 3/4, 0.5%) [7].

### Hematologic and nonhematologic AEs: postmarketing surveillance experience

In general, data from ruxolitinib postmarketing surveillance studies have been consistent with those reported in clinical trials. Overall, 14,445 patients received ruxolitinib treatment cumulatively since the Development International Birth Date (February 29, 2008) in Novartis- and Incyte-sponsored interventional clinical trials and managed access programs. Following 10 years of postmarketing use as of February 22, 2022, the cumulative estimated ruxolitinib exposure was 256,223 patient treatment years (PTY). A total of 127,349 AEs were reported from postmarketing data sources (spontaneous reports, postmarketing safety studies and registries, and literature cases), the majority of which were nonserious (63%; n = 79,690). The most frequently reported AEs were anemia (2.8%), fatigue (2.4%), hemoglobin decreased (2.3%), platelet count decreased (2.3%), and thrombocytopenia (1.8%). It is important to note the limitations of postmarketing surveillance data, which are collected under less rigorous conditions than clinical trials, have varied reporting rates over time, and require assumption of causality for regulatory reporting. Nevertheless, the postmarketing safety data for ruxolitinib are generally consistent with what has been reported in multiple randomized controlled trials [2, 3]. 

### Influence of disease stage and treatment delay on AEs

Timing of ruxolitinib treatment introduction—either based on disease stage, defined by International Prognostic Scoring System (IPSS) risk status (categorized by age, hemoglobin level, leukocyte count, circulating blasts, and constitutional symptoms [19]), or time to intervention from MF diagnosis—can contribute to AE variability. COMFORT-I and COMFORT-II were limited to patients with IPSS risk status of intermediate-2 or high [2, 3]; however, additional studies of ruxolitinib in patients with MF enrolled intermediate-1 patients, including JUMP (n = 163) [20] and a multicenter Italian study (n = 70) [21]. Cross-study comparisons suggest that IPSS intermediate-1 patients experience fewer toxicities compared with patients with higher-risk disease. Grade 3/4 anemia in patients treated with ruxolitinib ranged from 42–45% in the COMFORT studies compared with 22–25% in studies with intermediate-1 patients; corresponding ranges for grade 3/4 thrombocytopenia were 8–13% and 3–11%, respectively [2, 3, 20, 21].

The timing of ruxolitinib treatment initiation on the occurrence of AEs in MF was evaluated in a pooled analysis of COMFORT-I and -II (N = 525) [4]. Patients who initiated ruxolitinib treatment earlier (≤ 12 vs > 12 months after diagnosis) tended to be younger, had less severe cytopenias at baseline, and had fewer thrombocytopenia and anemia events over the course of treatment. Earlier treatment was also associated with better efficacy outcomes, including spleen volume response and OS.

## Managing anemia in patients treated with ruxolitinib

Guidance for managing anemia in MF patients treated with ruxolitinib comes primarily from the COMFORT trials, most commonly via the use of red blood cell transfusions. In COMFORT-I, 60% of patients receiving ruxolitinib and 38% of placebo recipients received red blood cell transfusions (mean, 1.7 and 2.2 transfusions per month, respectively), likely contributing to the low discontinuation rates for anemia (< 1%) [1, 3]. In JUMP, the number of transfusion-dependent patients at baseline (7.1%) who received red blood cell transfusions during the study was highest during the first 12 weeks of treatment and decreased over time [20]. Ruxolitinib can also be combined with several secondary treatments for anemia, which is an area of intense research interest. Erythropoiesis-stimulating agents, anabolic steroids such as danazol, the erythroid maturation agent luspatercept, and immunomodulatory imide agents such as thalidomide may provide clinical benefit [5, 22–25]. For example, in JUMP, 19.1% of patients received concomitant erythropoiesis-stimulating agents to manage anemia [20].

**Fig. 2:**
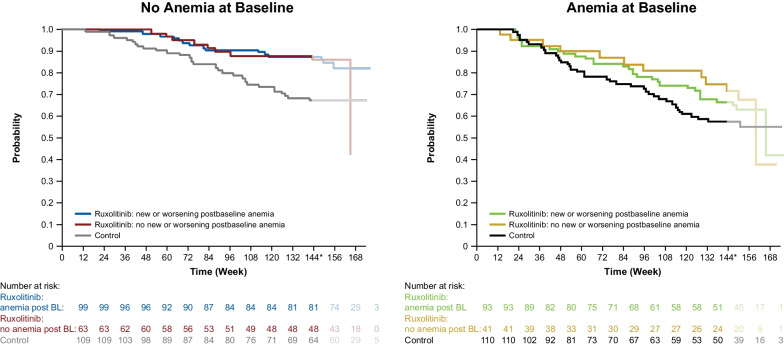
Overall survival by anemia status and treatment in the COMFORT trials. Gupta et al. [28]. Reprinted with permission. Copyright (2016) Ferrata Storti Foundation. *BL* baseline, *Rux* ruxolitinib

The results from multiple analyses have shown that baseline anemia is not a contraindication for ruxolitinib use. In COMFORT-I, patients in the ruxolitinib arm who experienced new-onset grade 3/4 anemia had similar improvements in symptoms and reductions in spleen volume as those without anemia [3]. Furthermore, in pooled analyses of COMFORT data, ruxolitinib provided an OS benefit versus control treatment regardless of anemia status at baseline or development of anemia during study treatment (Fig. 2) [26–28]. In addition, among patients treated with ruxolitinib, OS was not affected by transfusion status (transfusion dependent vs not dependent; transfusion independent vs not independent) at Week 24 [26]. Notably, development of anemia during treatment with ruxolitinib in the COMFORT trials was not prognostic for OS [27, 28]. These findings from clinical trial settings have also been observed in a real-world analysis, which showed that decreases in hemoglobin 6 months after ruxolitinib initiation did not affect OS [29].

Given the high frequency of anemia in MF, the phase 2 REALISE study evaluated an alternative ruxolitinib dosing strategy in 51 anemic (hemoglobin < 10 g/dL) patients with MF (Table 2) [9]. Patients received a starting dose of ruxolitinib of 10 mg twice daily [bid] with dose increases permitted based on platelet count and spleen response after 12 weeks of therapy [9]. Any-grade and grade ≥ 3 worsening of anemia were reported by 35% and 31% of patients, respectively, which were lower rates than those reported in the COMFORT studies and JUMP despite the majority of patients in these trials not having anemia at baseline [2, 7, 30]. Overall, the dosing regimen was well tolerated and efficacious, and spleen responses were observed regardless of transfusion dependence.

**Table 2:** Practical considerations for ruxolitinib dosing

Anemia (hemoglobin < 10 g/dL)	As a first step, rule out coexisting causes of anemia (e.g., gastrointestinal bleeding, hemolysis, or deficiencies in iron, vitamin B12, or folate) [5] Based on the REALISE study, initiate ruxolitinib at 10 mg bid. After 12 weeks, increase dose as required to achieve ≥ 50% reduction in spleen length vs baseline (at 12 weeks, increase dose to 15 mg if platelet counts ≥ 100 × 10^9^/L; at subsequent 4-week intervals, increase dose at 5-mg bid intervals up to 25 mg bid if platelet counts ≥ 200 × 10^9^/L) [9] Manage with ruxolitinib dose modifications/interruptions and RBC transfusions [1, 5]; avoid ruxolitinib discontinuation when possible Additional management options that can be considered alone or alongside ruxolitinib therapy include [5]: Erythropoiesis-stimulating agents if serum erythropoietin < 500 mU/mL [22] Anabolic steroid treatment or immunomodulatory imide agents, including in transfusion-dependent patients [25, 63]
Platelet counts [1, 48]	Starting doses of ruxolitinib are platelet-count dependent and vary by geographic region [1, 48] > 200 × 10^9^/L: ruxolitinib 20 mg bid 100–200 × 10^9^/L: ruxolitinib 15 mg bid 75– < 100 × 10^9^/L: ruxolitinib 5–10 mg bid* 50– < 75 × 10^9^/L: ruxolitinib 5 mg bid Alternate dosing strategy^†^ > 400 × 10^9^/L: ruxolitinib 20 mg bid 200–400 × 10^9^/L: ruxolitinib 15 mg bid 100–200 × 10^9^/L: ruxolitinib 10 mg bid < 100 × 10^9^/L: ruxolitinib 5 mg bid; uptitrate dosing to ≥ 10 mg bid
CYP3A4 inhibitors^‡^	Reduce starting ruxolitinib dose when used in combination with strong CYP3A4 inhibitors including fluconazole [1]
Renal impairment	Modify ruxolitinib dose in patients with moderate/severe renal impairment and those with end-stage renal disease [1]
Hepatic impairment	Reduce starting ruxolitinib dose in patients with hepatic impairment [1]
Ruxolitinib dose unintentionally exceeded	Appropriate supportive treatment for expected myelosuppression is recommended in cases where ruxolitinib dose is unintentionally exceeded; although hemodialysis is not expected to enhance elimination of ruxolitinib, patients can be managed for expected myelosuppression [1] A single dose of ruxolitinib may be administered following hemodialysis, using appropriate dose and monitoring thereafter
COVID-19	Discontinuing ruxolitinib due to SARS-CoV-2 infection or COVID-19 treatment is not advised [61, 62] Increased risk of death has been reported among patients with MF who discontinued ruxolitinib due to COVID-19 infection, indicating that continuing treatment with ruxolitinib may be advisable in this situation [62]

*bid* twice daily, *CYP3A4* cytochrome P450 3A4, *RBC* red blood cell

^*^Recommended ruxolitinib dosing for patients with platelet counts 75– < 100 × 10^9^/L varies by region: US, 5 mg bid; EU, 10 mg bid [1, 48]

^†^Per standard practice of author John Mascarenhas

^‡^Additional information regarding dosing in patients with CYP3A4 inhibitors can be found in the ruxolitinib US prescribing information [1]

## Managing thrombocytopenia in ruxolitinib-treated patients

Table 2 outlines practical considerations for ruxolitinib dosing for patients with comorbidities, including thrombocytopenia, anemia, and COVID-19.

Support for ruxolitinib dosing in patients with thrombocytopenia comes largely from EXPAND and a phase 2 study. EXPAND evaluated ruxolitinib in 69 patients with MF and low platelet counts (50– < 100 × 10^9^/L) and showed that platelet counts decreased initially during the first few weeks of treatment before stabilizing over time [8]. The phase 2 study evaluated ruxolitinib (starting dose, 5 mg bid) in 66 patients with low platelets (50– < 100 × 10^9^/L), reporting that platelet counts remained relatively stable through Week 24 [31]. EXPAND, as well as JUMP, demonstrated that patients with thrombocytopenia benefit from ruxolitinib treatment, including spleen response and symptom improvements [7, 8].

Although platelet transfusions may help manage thrombocytopenia in some patients [5, 32], currently there is limited evidence to support other treatments for thrombocytopenia. Findings from a small number of patients suggest danazol and pegylated interferon alpha may increase platelet counts in thrombocytopenic patients with MF [32]; for example, platelet increases with pegylated interferon were observed in five of eight thrombocytopenic patients, whereas nine of 62 (15%) patients in the overall study cohort developed thrombocytopenia [33]. In a separate combination study with ruxolitinib and pegylated interferon, three of 18 (17%) patients developed grade 1/2 thrombocytopenia, although patients with platelet counts < 100 × 10^9^/L were excluded from the study [34]. Conversely, a lack of platelet response to the nonpeptide thrombopoietin receptor agonist eltrombopag was reported in six patients with MF with persistent thrombocytopenia who were treated with ruxolitinib in a nonrandomized, single-arm phase 2 study [35].

## Risk of serious AEs

Although oral ruxolitinib is not accompanied by a boxed warning, the FDA recently added a boxed warning to all JAK inhibitors indicated for the treatment of arthritis and other inflammatory conditions. This was driven primarily by findings from the postmarketing study of tofacitinib versus tumor necrosis factor (TNF) blockers in rheumatoid arthritis [36–38]. The boxed warning has since been extended to the topical formulation of ruxolitinib approved for the treatment of mild to moderate atopic dermatitis and nonsegmental vitiligo [36]. Events flagged by the FDA in these inflammatory conditions include increased risk of serious infections, major adverse cardiovascular events (MACE), lymphoma and other malignancies, and thrombosis [36, 39].

### Infections

A meta-analysis of clinical trial publications of ruxolitinib in patients with myeloproliferative neoplasms (MPNs) demonstrated that the risk of overall infection was not elevated with ruxolitinib treatment, including in COMFORT-I and -II [40], and was typically lower than rates reported in a Swedish population with MPNs [41]. In JUMP, infection rates were low, predominantly grade 1/2, and led to ruxolitinib discontinuation in few (2.6%) patients (Table 1) [7]. The most common infections were pneumonia (any grade, 7.3%; grade 3/4, 4.7%), urinary tract infection (5.9%; 1.2%, respectively), herpes zoster (5.2%; 0.5%), and nasopharyngitis (5.2%; 0%); tuberculosis was reported in 0.2% of patients. ROBUST, EXPAND, and REALISE similarly reported that infection rates were low and were predominantly grade 1/2 [6, 8, 9].

Herpes zoster infections have been reported with ruxolitinib. In the 5-year COMFORT-I analysis, most cases of herpes zoster were single episodes that were grade ≤ 2 and resolved without long-term sequelae. By comparison, as noted above, herpes zoster was reported in 5.2% of patients (grade 3/4, 0.5%) in JUMP [7] and 3.5% (grade 3/4, 0%) in a real-world study in patients with intermediate-risk MF [14]. Use of non-live, varicella zoster subunit vaccine to prevent herpes zoster should be considered for patients receiving ruxolitinib [5], although vaccination may be reserved for older and/or more frail patients; it should be noted that this subunit vaccine was not yet available when COMFORT-I or JUMP were conducted.

Cases of sepsis were also observed in the COMFORT studies. A 5-year analysis of data from COMFORT-I reported that rates of grade 3/4 sepsis were 1.7 and 1.5 events/100 patient-years of exposure (PYE) in the ruxolitinib randomized and ruxolitinib crossover groups, respectively, and 1.0/100 PYE during the 24-week placebo treatment period [15]. Grade 3/4 sepsis was also reported in 1.5% of patients in JUMP [7] and in 4.2% in ROBUST [6].

### Infections: postmarketing surveillance experience

The rates of serious infections reported during postmarketing surveillance have been similar to those observed during clinical studies, and no new types or patterns of serious infections have been identified during 10 years of postmarketing data collection.

### Cardiovascular events

As described above, a clinical trial in patients with rheumatoid arthritis reported an increased risk of MACE (defined as cardiovascular death, myocardial infarction, or stroke) for patients receiving tofacitinib compared with those receiving TNF blockers [38]; this led to the inclusion of a boxed warning on all JAK inhibitors indicated for the treatment of arthritis and other inflammatory conditions. In a 5-year follow-up of COMFORT-I in which patients were stratified by duration of treatment subgroups, grade 3/4 congestive heart failure was observed in 6.2% of patients treated with ruxolitinib for ≥ 48 months (0–1.1% among patients treated for < 48 months); grade 3/4 myocardial infarction was observed in 0–2.7% of patients, with no apparent effect of treatment duration (Table 1) [15]. In JUMP, in patients with a median (range) age of 67.0 (18–89) years, cardiac failure was reported as a serious AE in 1.9% of patients, and congestive cardiac failure was the primary cause of death in < 0.1% of patients [7]. Other cardiac-related causes of death were cardiac arrest (0.6%), cardiac failure (0.5%), cardiorespiratory arrest (0.3%), myocardial infarction and cardiogenic shock (both 0.2%), and cardiac disorder (< 0.1%).

Data from the four pivotal randomized clinical trials that led to FDA approval of ruxolitinib for MF and PV indications were evaluated using the FDA definition for MACE. Overall, the exposure-adjusted incidence rates of MACE in the ruxolitinib and control arms were similar across these four studies as well as in the pooled population (Table 3), despite much longer exposure to ruxolitinib compared with the limited duration of exposure during the control periods. In addition, a disproportionality analysis using the ruxolitinib global safety database, the FDA Adverse Event Reporting System (AERS) database, and the World Health Organization (WHO) VigiBase database showed no disproportionality reporting for MACE with ruxolitinib (Additional file 1: Tables 1 and 2). Taken together, these data do not indicate that patients with MF or PV who receive ruxolitinib have an increased risk of MACE.

**Table 3:** Incidence rate of MACE

Study/Disease State	Exposure-adjusted incidence rate of MACE, n/PY (rate per 100 PY)			
	Ruxolitinib arm	Control arm		
		Control + crossover to ruxolitinib	Before or without crossover	After crossover to ruxolitinib
COMFORT-I (MF)	7/460.4 (1.52)	6/353.8 (1.70)	1/98.9 (1.01)	6/254.9 (2.35)
COMFORT-II (MF)	7/409.5 (1.71)	2/146.9 (1.36)	1/67.2 (1.49)	1/79.7 (1.25)
RESPONSE (PV)	3/428.4 (0.70)	4/404.6 (0.99)	1/74.6 (1.34)	3/329.9 (0.91)
RELIEF (PV)	0/79.8 (0)	2/91.6 (2.18)	0/23.0 (0)	2/68.5 (2.92)
Pooled population	17/1378.1 (1.23)	14/996.9 (1.40)	3/263.8 (1.14)	12/733.1 (1.64)

MACE, major adverse cardiovascular event; MF, myelofibrosis; PV, polycythemia vera; PY, patient-year

Another complication of MF is pulmonary arterial hypertension (PAH), a relatively uncommon event that generally occurs in patients with advanced disease [42, 43]. Although exacerbation of PAH in a patient receiving ruxolitinib and panobinostat was previously observed in a single case report [44], another study in 15 patients with MF-associated PAH reported that treatment with ruxolitinib led to improvements in PAH in two-thirds of patients [45]. Data from a recent study showed that the JAK2 pathway is involved in PAH disease progression and that blocking this pathway with ruxolitinib led to improved cardiopulmonary function in preclinical PAH models [46]. Additional study may be warranted to further investigate this approach.

### Nonmelanoma skin cancers

Although nonmelanoma skin cancers (NMSCs) have been observed in patients treated with ruxolitinib (Table 1), in the 5-year analysis of COMFORT-I, NMSC occurred at similar rates between patients receiving ruxolitinib and placebo [15]. Rates of basal cell carcinoma were 2.7 per 100 PYE in the ruxolitinib-randomized group, 4.0 in the ruxolitinib crossover group, and 3.9 among patients during treatment with placebo; rates of squamous cell carcinoma of the skin were 1.9, 1.1, and 1.0, respectively [15]. Among patients treated with ruxolitinib in JUMP, NMSCs were the most common secondary malignancies and were reported in 2.7% of patients treated with ruxolitinib [7]. By comparison, a recent systematic review of patients with MPNs treated with HU in observational studies reported NMSC rates of 0.29% (10/3411 patients in a retrospective Italian study; median age, ≈63 years), 9.6% (127/1316 in an Italian case-control study; median HU exposure, 3 years), 13.6% (9/66 in a retrospective Czech study; median age, 64 years), and 34.2% (51/149 patients in a retrospective Australian study; median HU exposure, 4 years; median age, 66 years) [47]. Finally, in a noninterventional postauthorization safety study of 462 patients with MF that included prevalent or new users of ruxolitinib, a nonsignificant trend toward increased risk of NMSC with increasing ruxolitinib exposure was observed (hazard ratio [HR] corresponding to risk with each additional year of ruxolitinib, 1.2 [95% CI 0.9, 1.6]) [16]. The US prescribing information and European summary of product characteristics (SmPC) both mention that NMSCs have occurred in patients treated with ruxolitinib and recommend that patients have periodic skin examinations [1, 48], with the SmPC also stating: “Most of the MF and PV patients had histories of extended treatment with hydroxyurea and prior NMSC or pre-malignant skin lesions. A causal relationship to ruxolitinib has not been established.” 

### NMSC: postmarketing surveillance experience

In an analysis of the ruxolitinib global safety database encompassing 10 years of postmarketing use through February 22, 2022, the reporting rate for NMSC was 0.46 cases per 100 PYE. No new findings were identified compared with the results seen in the clinical trials. The clinical trial and postmarketing surveillance data are consistent with the recommendations in the US prescribing information and European SmPC [1, 48], with no conclusive evidence supporting a causal relationship between ruxolitinib use and NMSCs.

### Lymphoma and other malignancies

Cases of lymphoma and other malignancies have been reported in patients with MF treated with ruxolitinib. Second malignancies were reported by 6.1% of patients treated with ruxolitinib in JUMP [7]; four patients (0.2%) developed lymphomas, including non-Hodgkin lymphoma (n = 2), B cell lymphoma (n = 1), and lymphoma (n = 1) [7]. Notably, the rates were lower in JUMP than in a Swedish population-based study of patients with MPNs who were not exposed to ruxolitinib (lymphoma, 1%; acute myeloid leukemia, 3.0%) [7, 41].

In the postauthorization safety study described above, malignancies were reported at a rate of 10.1 events per 100 PYE among prevalent ruxolitinib users (n = 259) and 7.4 events per 100 PYE among new ruxolitinib users (n = 32) [16]. Overall, five cases of lymphoma were reported (all prevalent ruxolitinib users), including diffuse large B cell lymphoma (n = 3), B cell small lymphocytic lymphoma (n = 1), and mycosis fungoides (n = 1). 

### Lymphoma and other malignancies: postmarketing surveillance experience

A disproportionality analysis using the ruxolitinib global safety database, the FDA AERS database, and the WHO VigiBase database was performed for ruxolitinib and second primary malignancies in postmarketing data. The results showed no disproportionality for malignancies with ruxolitinib in these databases (Additional file 1: Tables 3, 4).

### Other AEs of interest based on the JAK inhibitor mechanism of action

Although progressive multifocal leukoencephalopathy (PML) is primarily associated with the use of monoclonal antibodies, and sporadic incidence of multifocal leukoencephalopathy has been reported in patients with hematologic malignancies [49], isolated cases have been reported following treatment with JAK inhibitors [50–52]. Encephalopathy has also been observed with the use of the JAK2 inhibitor fedratinib for MF: four cases (2%) of confirmed or suspected Wernicke encephalopathy were observed in the phase 3 JAKARTA trial in patients treated with fedratinib 500 mg/d [53]. The molecular basis for occurrence of Wernicke encephalopathy in patients receiving fedratinib appears to be related to inhibition of thiamine uptake, which is a mechanistic feature of fedratinib not present in other marketed JAK inhibitors [54]. In a later analysis of the JAKARTA trial, it was reported that no patients receiving fedratinib 400 mg/d experienced Wernicke encephalopathy [55].

In a natural history study of 100 patients treated with the JAK1/JAK2 inhibitor momelotinib in phase 1/2 clinical studies at the Mayo Clinic, 44% developed treatment-emergent peripheral neuropathy (primarily grade 1, but not reversible in most patients) [56] compared with 10–11% of momelotinib-treated patients in the phase 3 SIMPLIFY trials [57, 58]. Peripheral neuropathy was not observed in the clinical trials of ruxolitinib in MF [7, 12, 26].

### Other AEs of interest based on the JAK inhibitor mechanism of action: postmarketing surveillance experience

As of February 22, 2022, with approximately 256,223 PTY of ruxolitinib exposure, there have been only three confirmed cases of PML and no cases of Wernicke encephalopathy. Based on these findings, there is no evidence of association between ruxolitinib and either PML or Wernicke encephalopathy. This observation is similar to the pre-ruxolitinib era, where PML was a sporadic finding in MPN patients.

### Deaths

In a pooled analysis of COMFORT-I and -II, risk of death was reduced by 30% among patients randomized to ruxolitinib compared with controls who received placebo (COMFORT-I) or best available care (COMFORT-II) [26]; median OS was 5.3 years for ruxolitinib versus 3.8 years for control [26]. In COMFORT-I, the most common AEs resulting in death in the ruxolitinib randomized arm were sepsis (2.6%), disease progression (1.9%), and pneumonia (1.9%). In COMFORT-II, none of the causes of death occurring on treatment or within 28 days of treatment discontinuation occurred in > 1 patient.

Ongoing surveillance and signal detection from cumulative postmarketing data (both aggregate disproportionality analysis and individual case review) have not identified increased risk of death for specific AEs. Postmarketing reports of death with information sufficient for medical assessment predominantly reflect underlying diseases or recognized comorbidities of MF. However, unlike COMFORT-I and -II with long-term follow-up [26], the lack of reliable denominators or systematic data collection does not allow meaningful survival rates or AE rates to be generated from postmarketing data.

## Symptom exacerbation following interruption or discontinuation of ruxolitinib treatment

Symptom exacerbation following interruption or discontinuation of treatment with ruxolitinib has been reported in some patients [59], for whom symptoms from MPNs may return to pretreatment levels over a certain time period. It should be noted that this condition refers to the reemergence of MF symptoms, not to the appearance of new AEs or a rebound in symptoms above baseline. This condition was examined in some detail in a real-world survey conducted in 22 academic hematology centers that included 251 patients with MF who discontinued ruxolitinib. Treatment failure (including lack or loss of response or leukemic transformation) was the leading cause of ruxolitinib stoppage (61%), followed by AEs (29%). In this study, ruxolitinib was gradually decreased before discontinuation in 35% of patients and was abruptly stopped in the remaining patients. Symptom exacerbation following ruxolitinib discontinuation occurred in 34 (14%) patients and appeared quickly (median time, 7 days post-discontinuation). Symptom exacerbation was mild (no intervention required) in 21/251 (8.4%) patients, moderate (medical interventions required, including restarting ruxolitinib or administration of steroids or oral analgesics) in 10/251 (4.0%), and severe (intravenous medications, hospital admission, splenectomy, or delaying hematopoietic allogeneic transplantation required) in 3/251 (1.2%). In a multivariable Cox regression analysis, only platelet count < 100 × 10^9^/L (HR, 2.98 [95% CI 1.29, 6.90]) and spleen ≥ 10 cm below costal margin at ruxolitinib stoppage (HR, 2.03 [95% CI 1.01, 4.17]) were associated with a higher probability of symptom exacerbation following ruxolitinib discontinuation; no association was identified with age ≥ 70 years, sex, secondary MF, hemoglobin < 10 g/dL, use of ruxolitinib tapering before discontinuation, ruxolitinib dose ≤ 10 mg bid, Myelofibrosis Symptom Assessment Form (MFSAF) total symptom score ≥ 20, or Charlson Comorbidity Index ≥ 2.

The pathophysiology of symptom exacerbation following ruxolitinib discontinuation or interruption is not well understood, but it is theorized to result from a rapid rebound of inflammatory cytokines that were suppressed under ruxolitinib treatment [60]. Guidelines for managing symptom exacerbation following ruxolitinib discontinuation or interruption are described in the US prescribing information [1]. In our practices, we recommend reinstatement of ruxolitinib in patients showing signs of a rebound, as well as administration of steroids. A pulsed dose of prednisone during the tapering period can help offset rebound in patients receiving ruxolitinib doses > 10 mg bid, those who were very symptomatic before ruxolitinib initiation, or those with splenomegaly if not transitioning the patient to another JAK inhibitor.

### Tapering strategies

The US prescribing information for ruxolitinib recommends a gradual tapering of ruxolitinib (e.g., dose reductions of 5 mg bid each week) for reasons other than cytopenias [1]. However, there are no consensus guidelines providing specific recommendations for ruxolitinib taper. The National Comprehensive Cancer Network (NCCN) recommends gradual taper when discontinuing or interrupting therapy [5], whereas real-world studies have shown that tapering patterns are highly variable across centers, with dose reductions of 5 or 10 mg/d and dose reduction intervals ranging from every 3 to 30 days [59].

## Practical considerations

Table 2 includes a list of practical considerations related to ruxolitinib dosing, including information from the US prescribing information, European SmPC, and real-world experience with ruxolitinib. Of particular note in the COVID-19 era, it is not advisable to discontinue ruxolitinib due to SARS-CoV-2 infection or COVID-19 treatment. This recommendation is based on an increased risk of death reported in patients with MF who discontinued ruxolitinib treatment due to COVID-19 infection [61, 62]. In an observational retrospective study promoted by the European LeukemiaNet, a multivariable analysis identified an 8.5-fold increased risk of death among patients with MF and COVID-19 who discontinued ruxolitinib compared with those who continued treatment [62].

## Conclusions

The safety profile of ruxolitinib in MF has been established in the COMFORT trials and during the 10 years since its approval by the FDA, including in a series of clinical trials, pooled analyses, expanded-access studies, and postmarketing analyses. Hematologic AEs, which are to be expected given the mechanism of action as a JAK1/JAK2 inhibitor, are generally manageable with dose modifications, transfusions, or both; nonhematologic AEs were generally observed at a similar rate to placebo or best available therapy in the COMFORT trials. Importantly, presence of anemia is not a contraindication for ruxolitinib use and is not prognostic for response to ruxolitinib treatment. With ruxolitinib remaining the standard of care in MF more than a decade after its initial approval, the importance of this agent in the MF armamentarium is clear. Future analyses may shed further light on the ruxolitinib safety profile, including dose optimization strategies for ruxolitinib treatment interruptions or discontinuations and use in combination regimens.

## Supplementary Information

**Additional file 1.** Supplemental disproportionality analyses.

## Data Availability

Incyte Corporation (Wilmington, DE, USA) is committed to data sharing that advances science and medicine while protecting patient privacy. Qualified external scientific researchers may request anonymized datasets owned by Incyte for the purpose of conducting legitimate scientific research. Researchers may request anonymized datasets from any interventional study (except Phase 1 studies) for which the product and indication have been approved on or after January 1, 2020 in at least one major market (e.g., US, EU, and JPN). Data will be available for request after the primary publication or 2 years after the study has ended. Information on Incyte’s clinical trial data sharing policy and instructions for submitting clinical trial data requests are available at: https://www.incyte.com/Portals/0/Assets/Compliance%20and%20Transparency/clinical-trial-data-sharing.pdf?ver=2020-05-21-132838-960.
