# Supplementary material for: Ten years of treatment with ruxolitinib for myelofibrosis: a review of safety
Source: J Hematol Oncol. 2023 Jul 27;16:82. doi: 10.1186/s13045-023-01471-z (PMC10373260; doi:10.1186/s13045-023-01471-z)
Supplement: Supplementary file 1 — Additional file 1. Supplemental disproportionality analyses. [file 13045_2023_1471_MOESM1_ESM.docx]

**Ten Years of Treatment With Ruxolitinib for Myelofibrosis: A Review of Safety**

Srdan Verstovsek,^1^ Ruben A. Mesa,^2^ Robert A. Livingston,^3^ Wilson Hu,^3^ John Mascarenhas^4^

^1^The University of Texas MD Anderson Cancer Center, 1515 Holcombe Blvd, Houston, TX 77030, USA; ^2^Atrium Health Wake Forest Baptist Comprehensive Cancer Center, Medical Center Blvd, 11th Floor, Wake Forest University School of Medicine, Winston Salem, NC 27157, USA; ^3^Incyte Corporation, 1801 Augustine Cut-Off, Wilmington, DE 19803, USA; ^4^Icahn School of Medicine at Mount Sinai, 1470 Madison Avenue, New York, NY 10029, USA

**Supplementary Materials**

**Supplementary Table 1. Disproportionality analysis of MACE in the ruxolitinib global safety database**

| **Event** | **Total case count*** | **EB05^†^** |
| --- | --- | --- |
|  | | |
| Myocardial infarction | 201 | 0.79 |
| Acute myocardial infarction | 25 | 0.51 |
| Coronary artery thrombosis | 1 | 0.08 |
| Postprocedural myocardial infarction | 1 | 0.25 |
| Stroke | | |
| Cerebrovascular accident | 243 | 0.86 |
| Cerebral infarction | 18 | 0.26 |
| Ischemic stroke | 16 | 0.40 |
| Cerebral thrombosis | 5 | 0.38 |
| Hemorrhagic stroke | 5 | 0.22 |
| Cerebral artery embolism | 2 | 0.30 |
| Embolic stroke | 2 | 0.17 |
| Lacunar infarction | 1 | 0.09 |
| Cerebellar infarction | 1 | 0.01 |
| Carotid artery thrombosis | 1 | 0.14 |
| Thrombotic stroke | 1 | 0.18 |

EB, empirical Bayesian; MACE, major adverse cardiovascular event.

* Case counts comprised spontaneous reports only until cutoff December 2021.

^†^ EB05 ≤2 indicates no disproportionality.

Supplementary Table 2. Disproportionality analysis of MACE in WHO VigiBase and FDA AERS databases

| Event | VigiBase (data to Q4 2021) | | | | AERS (data to Q3 2021) | | | |
| --- | --- | --- | --- | --- | --- | --- | --- | --- |
|  | **Total case count*** | **EBGM** | **EB05** | **EB95** | **Total case count^†^** | **EBGM** | **EB05** | **EB95** |
| MI | 211 | 1.019 | 0.909 | 1.14 | 260 | 0.757 | 0.683 | 0.837 |
| Stroke | 274 | 0.964 | 0.872 | 1.063 | 344 | 0.747 | 0.683 | 0.815 |
| Other cardiovascular deaths (excluding MI and stroke) | | | | | | | | |
| Heart failure | 84 | 2.048 | 1.707 | 2.44 | 234 | 2.054 | 1.843 | 2.284 |
| PVD | 1 | 0.94 | 0.238 | 2.773 | 11 | 1.236 | 0.754 | 1.934 |
| Pericardial hemorrhage fatal | — | — | — | — | 2 | 1.265 | 0.464 | 2.914 |
| Cardiac and vascular procedural complications fatal | — | — | — | — | 1 | 0.489 | 0.143 | 1.311 |
| Myocardial hemorrhage fatal | — | — | — | — | — | — | — | — |
| Sudden cardiac death | 2 | 0.532 | 0.18 | 1.295 | 3 | 0.465 | 0.195 | 0.972 |

AERS, Adverse Event Reporting System; EBGM, empirical Bayes geometric mean; EBXX, empirical Bayes XXth percentile; FDA, US Food and Drug Administration; MACE, major adverse cardiovascular event; MI, myocardial infarction; PVD, peripheral vascular disease; WHO, World Health Organization.

* Data through to Q4 2021.

^†^ Data through to Q3 2021.

**Supplementary Table 3. Disproportionality analysis of malignant tumors in the ruxolitinib global safety database**

| **Event*** | **Total case count^†^** | **EB05^‡^** |
| --- | --- | --- |
| Malignant tumors | 2730 | 1.37 |
| Hematologic malignant tumors | 1206 | 2.42^§^ |
| Nonhematologic malignant tumors | 1621 | 1.03 |
| Malignant lymphomas | 168 | 0.98 |

EB, empirical Bayesian; MedDRA, Medical Dictionary for Regulatory Activities; MPN, myeloproliferative neoplasm.

* Standard MedDRA query (narrow).

^†^ Case counts comprised spontaneous reports only until cutoff December 2021.

^‡^ EB05 ≤2 indicates no disproportionality.

^§^ EB05 >2.0 mainly because of leukemic transformation of underlying MPN.

Supplementary Table 4. Disproportionality analysis of malignant tumors in WHO VigiBase and FDA AERS databases

| Event* | VigiBase | | | | AERS | | | |
| --- | --- | --- | --- | --- | --- | --- | --- | --- |
|  | **Total case count^†^** | **EBGM** | **EB05^‡^** | **EB95** | **Total case count^†^** | **EBGM** | **EB05^‡^** | **EB95** |
| Malignant tumors | 2331 | 1.45 | **1.401** | 1.5 | 3144 | 1.076 | **1.045** | 1.108 |
| Hematologic malignant tumors | 967 | 4.681 | **4.439^§^** | 4.934 | 1435 | 3.704 | **3.546^§^** | 3.868 |
| Malignant lymphomas | 132 | 1.889 | **1.635** | 2.174 | 210 | 1.469 | **1.31** | 1.643 |
| Nonhematologic malignant tumors | 1445 | 1.018 | **0.975** | 1.063 | 1836 | 0.712 | **0.685** | 0.74 |

AERS, Adverse Event Reporting System; EBGM, empirical Bayes geometric mean; EBXX, empirical Bayes XXth percentile; FDA, US Food and Drug Administration; MedDRA, Medical Dictionary for Regulatory Activities; MPN, myeloproliferative neoplasm; WHO, World Health Organization.

* Standard MedDRA query (narrow).

^†^ Case counts comprised spontaneous reports only until cutoff December 2021.

^‡^ EB05 ≤2 indicates no disproportionality.

^§^ EB05 >2.0 mainly because of leukemic transformation of underlying MPN.
